# Correction: Chen et al. New n-p Junction Floating Gate to Enhance the Operation Performance of a Semiconductor Memory Device. *Materials* 2022, *15*, 3640

**DOI:** 10.3390/ma15196738

**Published:** 2022-09-28

**Authors:** Yi-Yueh Chen, Su-Jien Lin, Shou-Yi Chang

**Affiliations:** Department of Materials Science and Engineering, National Tsing Hua University, Hsinchu 30013, Taiwan

The authors would like to make corrections to a recently published paper [1].

## Removal of Authors

In consideration of the contributions to this work, the following authors are removed from the publication: Feng-Ming Lee, Yu-Yu Lin, Chih-Hsiung Lee, Wei-Chen Chen, Che-Kai Shu, and Chih-Yuan Lu. Their support is acknowledged as indicated in the Acknowledgements. The corrected authors are Yi-Yueh Chen, Su-Jien Lin, and Shou-Yi Chang.

## Removal of Affiliations

According to the change in authorship, the corrected affiliation appears below: 

Department of Materials Science and Engineering, National Tsing Hua University, Hsinchu 30013, Taiwan; sjlin@mx.nthu.edu.tw (S.-J.L.); changsy@mx.nthu.edu.tw (S.-Y.C.).

## Update of Author Contributions

According to the change in authorship, the corrected Author Contributions statement appears below:

Conceptualization, Y.-Y.C.; methodology, Y.-Y.C.; formal analysis, Y.-Y.C.; investigation, Y.-Y.C.; resources, Y.-Y.C.; data curation, Y.-Y.C.; writing—original draft, Y.-Y.C. and S.-Y.C.; writing—review & editing, Y.-Y.C. and S.-Y.C.; supervision, S.-J.L. and S.-Y.C.; project administration, Y.-Y.C.; funding acquisition, S.-J.L. All authors have read and agreed to the published version of the manuscript.

## Change of Acknowledgements

The corrected Acknowledgments statement appears below:

The authors gratefully acknowledge Macronix International Co., Ltd. for their support and technical discussions about this work.

The authors apologize for any inconvenience caused and state that the scientific conclusions are unaffected. This correction was approved by the Academic Editor. The original publication has also been updated.
